# In Vitro Differentiation of Human Skin-Derived Cells into Functional Sensory Neurons-Like

**DOI:** 10.3390/cells9041000

**Published:** 2020-04-17

**Authors:** Adeline Bataille, Raphael Leschiera, Killian L’Hérondelle, Jean-Pierre Pennec, Nelig Le Goux, Olivier Mignen, Mehdi Sakka, Emmanuelle Plée-Gautier, Cecilia Brun, Thierry Oddos, Jean-Luc Carré, Laurent Misery, Nicolas Lebonvallet

**Affiliations:** 1 EA4685 Laboratory of Interactions Neurons-Keratinocytes, Faculty of Medicine and Health Sciences, University of Western Brittany, F-29200 Brest, France; ade.bataille@gmail.com (A.B.); raphael.leschiera@univ-brest.fr (R.L.); killianlherondelle@gmail.com (K.L.); mehdisakka@hotmail.fr (M.S.); emmanuelle.plee-gautier@chu-brest.fr (E.P.-G.); jean-luc.carre@chu-brest.fr (J.-L.C.); laurent.misery@chu-brest.fr (L.M.); 2 EA 4324 Optimization of Physiological Regulation, Faculty of Medicine and Health Sciences, University of Western Brittany, F-29200 Brest, France; jpennec@univ-brest.fr; 3 INSERM UMR1227 B Lymphocytes and autoimmunity, University of Western Brittany, F-29200 Brest, France; nelig.legoux@orange.fr (N.L.G.); olivier.mignen@univ-brest.fr (O.M.); 4 Department of Biochemistry and Pharmaco-Toxicology, University Hospital of Brest, 29609 Brest, France; 5 Johnson & Johnson Santé Beauté France Upstream Innovation, F-27100 Val de Reuil, France; CBRUN1@its.jnj.com (C.B.); toddos@its.jnj.com (T.O.); 6 Department of Dermatology, University Hospital of Brest, 29609 Brest, France

**Keywords:** sensory neuron, adult stem cell, differentiation, TRP

## Abstract

Skin-derived precursor cells (SKPs) are neural crest stem cells that persist in certain adult tissues, particularly in the skin. They can generate a large type of cell in vitro, including neurons. SKPs were induced to differentiate into sensory neurons (SNs) by molecules that were previously shown to be important for the generation of SNs: purmorphamine, CHIR99021, BMP4, GDNF, BDNF, and NGF. We showed that the differentiation of SKPs induced the upregulation of neurogenins. At the end of the differentiation protocol, transcriptional analysis was performed on *BRN3A* and a marker of pain-sensing nerve cell *PRDM12* genes: 1000 times higher for *PRDM12* and 2500 times higher for *BRN3A* in differentiated cells than they were in undifferentiated SKPs. Using immunostaining, we showed that 65% and 80% of cells expressed peripheral neuron markers BRN3A and PERIPHERIN, respectively. Furthermore, differentiated cells expressed TRPV1, PAR2, TRPA1, substance P, CGRP, HR1. Using calcium imaging, we observed that a proportion of cells responded to histamine, SLIGKV (a specific agonist of PAR2), polygodial (a specific agonist of TRPA1), and capsaicin (a specific agonist of TRPV1). In conclusion, SKPs are able to differentiate directly into functional SNs. These differentiated cells will be very useful for further in vitro studies.

## 1. Introduction

Recently, many studies have described the possibility of producing in vitro sensory neurons (SNs) from stem cells. Different teams have described similar protocols to differentiate human embryonic stem cells (hESCs), human induced pluripotent stem cells (hiPSCs), or a cell line of human neural progenitor cells into SNs through three major steps: (i) differentiation into neural crest cells by inhibiting transforming growth factors beta (TGF-β)/SMAD signaling and bone morphogenetic protein (BMP) signaling by using SB431542 and Noggin, LDN-193189, or dorsomorphin, (ii) activation of Wnt/β-catenin and/or BMP pathways for neuronal differentiation through treatment with CHIR99021, or Wnt-1 and/or BMP-4, and (iii) maturation into SNs by treatment with neurotrophic factors, such as nerve growth factor (NGF), brain-derived neurotrophic factor (BDNF), neurotrophin 3 (NT3), and glial cell line-derived neurotrophic factor (GDNF) [1,2,3,4,5,6].

The skin is the largest organ of the human body and possesses various functions, such as protection, homeostasis maintenance, and sensory and thermoregulatory functions. It is an interface between the organism and the external environment [7]. It is a complex and highly regenerative tissue that includes several stem cells or precursor cells, such as epidermal stem cells [8], dermal mesenchymal stem cells [9], hair follicle stem cells [10], and precursors of neural crest cells [11].

In 2001, skin-derived precursor cells (SKPs) were described as a novel population of multipotent stem cells [12]. These stem cells can be isolated from adult skin [12,13] and possess the capacity of self-renewal. Several studies have demonstrated that a wide variety of cell types could be derived from SKPs, such as cells expressing neuronal, glial, osteoblastic, smooth muscle, chondrocytic, and melanocytic lineage markers [12,13,14,15,16,17]. Furthermore, they express some markers common to neural crest stem cells (NCSCs), such as the progenitor neural NESTIN [14], the low-affinity neurotrophin receptor p75 (P75NTR) and transcription factors, such as SOX9, SOX10, PAX3, SLUG, and SNAIL [14,15]. The undifferentiated state of NCSCs is maintained by the control of two pathways involving Wnt and BMP in an endogenous niche, which is the hair follicle [18].

SKPs are NCSCs that persist in certain adult tissues, particularly within the skin, and they are located in hair follicles [14,19]. NCSCs have the ability to generate much of the peripheral nervous system, in particular, SNs [20]. During development, NCSC fate depends on several signaling pathways, such as the hedgehog (HH), Wnt/B-catenin, and BMP pathways. The HH pathway is involved due to its mitogenic role in controlling the proliferation of neural progenitor cells. This pathway is initiated by sonic hedgehog (SHH), which is involved in neural development [21]. In addition, it has been shown that the Wnt/B-catenin pathway, which is highly expressed in hair follicles, regulates sensory neuronal differentiation [22]. This signaling pathway regulates Neurogenin1 (NGN1) and Neurogenin2 (NGN2) expression, and they themselves control neuronal differentiation factor (NEUROD) expression, which has been shown to play a role in vertebrate neurogenesis [23,24]. Therefore, expression of the *NGN* genes is determined by whether the SN profile is acquired, and that is dependent on the Wnt signaling pathway. Moreover, a second signaling pathway, the BMP pathway, is important once sensory neuronal differentiation begins. BMPs, in particular, BMP7 and BMP4, are important regulators of sensory development [25]. BMP4 functions in SN maturation and innervation. BMPs can regulate the acquisition of neuron dependence on neurotrophins, such as NT3, neurotrophin 4 (NT4), NGF, and BDNF, for their survival [26].

The aim of this study was to obtain SNs using easily accessible biologic materials, such as skin, without using iPSCs or embryonic stem cells. We evaluated the possibility of obtaining SNs directly from SKPs. Here, we report a protocol for generating functional SNs from SKPs and neural crest cells. To achieve this goal, we used and adapted a protocol already established by Reinhardt et al., for the growth of hESCs and hiPSCs [5]. Differentiation was observed and characterized either by immunostaining or quantitative polymerase chain reaction (qPCR) or both. Overall, several markers were used for neural progenitor characterization, such as SOX1, NESTIN, SOX2, ZIC1, PAX3 and 6. For neural crest cells, HNK1, AP2, P75NTR, and SOX9 were used as markers. Finally, to characterize the neuronal differentiation and peripheral neuronal profile, NGNs, PERIPHERIN, BRN3A, and PRDM12 were assessed. 

The SNs were characterized by immunochemistry and qPCR, and their functional maturation was evaluated by electrophysiology and calcium imaging.

## 2. Materials and Methods

All procedures followed were in accordance with the ethical standards of the responsible committee on human experimentation (institutional and national) and with the Helsinki Declaration of 1975, as revised in 2000. Informed consent was obtained from all patients for being included in the study.

### 2.1. Isolation and Cultivation of Human SKPs

**Figure 1:**
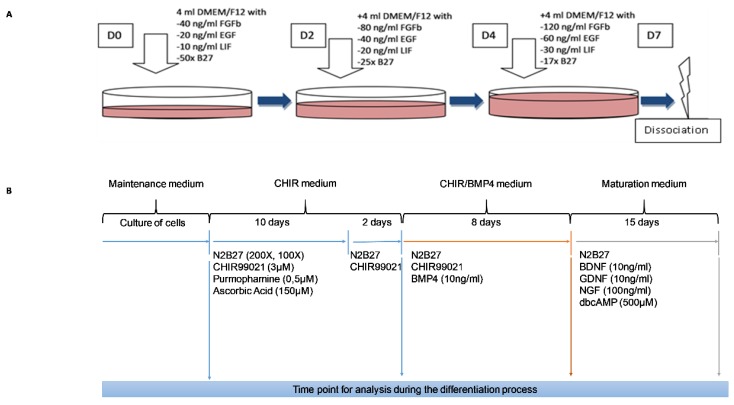
Schematic representation of the protocol used for the culture and differentiation of human skin-derived precursor cells (SKPs). Important factors are indicated for each step. **A**: Protocol for the selection and maintenance of SKPs. **B**: Protocol for the differentiation of SKPs.

Skin samples from 4 donors were used throughout the experiments. They were obtained following abdominal surgery. Written informed consent of no opposition was signed. SKPs were isolated using a slightly adapted protocol, as previously published [27]. Pieces of skin, 2 mm by 6 cm, were produced, and they were dissociated in 250 µg/mL Thermolysin (Sigma-Aldrich, Saint-Louis, MO, USA, T7902) overnight at 4 °C, followed by 2 h at 37 °C. Then, the epidermis and dermis were mechanically separated, placed together, and incubated in a 250 µg/mL collagenase IV (Sigma-Aldrich, Saint-Louis, MO, USA, C1889) solution for 3 h at 37 °C. After centrifugation at 700× *g* for 10 min, the supernatant was discarded, and the skin samples were finally dissociated by trypsin/EDTA (Lonza, Basel, Switzerland, BE17-161E) for 35 min at 37 °C. Following a second centrifugation, the pellet was resuspended in Dulbecco’s Modified Eagle Medium (DMEM; Lonza, Basel, Switzerland, BE12-604F) and then filtered through a 70 µm filter. These steps were performed twice. Finally, filtered cells were centrifuged at 90× *g* for 5 min and placed in a tissue culture Petri dish with maintenance medium. The SKP maintenance medium consisted of DMEM/F12 3/1 mixture (DMEM and DMEM/F12; Lonza, Basel, Switzerland, BE12-604F) with B27 50X (without vitamin A; Gibco, Thermo Fisher Scientific, Waltham, MA, USA, 12587-001), LIF (Leukemia Inhibitory Factor; Santa Cruz Biotechnology, Santa Cruz, CA, USA, sc-4377,) at 10 ng/mL, EGF (Epidermal growth factor; Sigma-Aldrich, Saint-Louis, MO, USA, E9644) at 20 ng/mL and FGF (Fibroblast growth factor; Sigma-Aldrich, Saint-Louis, MO, USA, F0291) at 40 ng/mL. Normocin (^1/500^, InvivoGen, San Diego, CA, USA, ANT-NR2) was added to all culture media. Twice a week, new medium was added to the culture media with an adjusted concentration of growth factors. In addition, every week, the medium was changed, and the cells were chemically dissociated using a dissociation kit (Stemcell technologies, Vancouver, Canada, Neurocult chemical dissociation kit 05707). This part of the protocol is shown in Figure 1A. After 1 month, some SKPs were adherent, and they were maintained as adherent cells in cell culture flasks.

### 2.2. Induction of SN Differentiation from Human SKPs

Cells were plated at a density of 12.10^3^ cells/cm^2^ in precoated poly-l-ornithine (15 µg/mL; Sigma-Aldrich, Saint-Louis, MO, USA, P3655)/laminin (1 µg/mL; Sigma-Aldrich, Saint-Louis, MO, USA, L2020)/fibronectin (50 µg/mL; Corning, NY, USA, 356008) dishes. They were expanded in the SKP maintenance medium, as previously described (Figure 1A), for a few days to reach 60% to 80% confluence before induction. To initiate differentiation into SNs, the SKP maintenance medium was replaced with DMEM/F12/Neurobasal 1/1/2 (Gibco, Thermo Fisher Scientific, Waltham, MA, USA, 21103-049) that contained B27 100X (without vitamin A), N2 200X (Gibco, Thermo Fisher Scientific, Waltham, MA, USA, 17502-048), 3 µM CHIR99021 (Sigma-Aldrich, Saint-Louis, MO, USA, SML1046), 0.5 µM PMA (Sigma-Aldrich, Saint-Louis, MO, USA, SML0868), and 150 µM ascorbic acid (Sigma-Aldrich, Saint-Louis, MO, USA, PHR1008). This medium was changed every day for 10 days. Then, for 2 days, PMA and ascorbic acid were removed (CHIR medium) from the medium. Following this differentiation into SNs, 10 ng/mL BMP4 (Sigma-Aldrich, Saint-Louis, MO, USA, SRP3016) was added to the medium already containing N2B27 and CHIR99021 (CHIR/BMP4 medium) for 8 days. For maturation of SNs, the medium that was used was composed of neurotrophic factors: 100 ng/mL NGF, 10 ng/mL GDNF, 10 ng/mL BDNF, and 500 µM dibutyryl cyclic AMP (dbcAMP; Sigma-Aldrich, Saint-Louis, MO, USA, D0260) (Maturation medium). This medium was changed every 2 days for 15 days. This part of the protocol is shown in Figure 1B.

### 2.3. Immunocytochemistry

Cultures were fixed and permeabilized with methanol for 5 min and then frozen. After drying, nonspecific binding sites were blocked with 10% goat serum and 1% bovine serum albumin (BSA; Sigma-Aldrich, Saint-Louis, MO, USA, A7906) in DPBS-T (^1/1000^ Tween 20) for 1 h at room temperature (RT). Cells were incubated with primary antibodies diluted in DPBS-T with 10% goat serum overnight at 4 °C. After being washed 3 × 5 min with DPBS-T, the cells were incubated at RT in the dark for 1.5 h with secondary antibodies incubated in DPBS-T. Then, the cells were washed 3 × 5 min and mounted with a hydrophilic liquid containing 4’-6-diamidino-2-phenylindole (Prolong Gold antifade reagent with DAPI, Thermo Fisher Scientific, Waltham, MA, USA, P3695). Stained cells were analyzed under a fluorescence microscope (Carl Zeiss, Oberkochen, Germany, Axiostar plus,). Pictures were taken with camera (Carl Zeiss, Oberkochen, Germany, AxioCam ICc1).

Cells counting and evaluation of each step of differentiation efficiency was performed using ImageJ software. Images of five random fields were taken from each well at a magnification of X20 or X10, such that a minimum of 30 cells/field could be observed. The ratio between the total number of positive cells for specific markers and the total number of DAPI-stained nuclei for each field was calculated.

The primary antibodies used in this study were rabbit anti-NESTIN (1:100, Abcam ab105389), rabbit anti-P75NTR (1:100, Abcam ab38335), rabbit anti-BRN3A (1:100, Abcam, Cambridge, UK, ab81213), mouse anti-PERIPHERIN (1:50, Abcam, Cambridge, UK, ab4573), mouse anti-CGRP (1:100, Abcam, Cambridge, UK, ab811887), rabbit anti-TRPA1 (1:200, Abcam, Cambridge, UK, ab58844), rabbit anti-SP (Substance P, 1:200, Sigma-Aldrich, Saint-Louis, MO, USA, S1542), rabbit anti-TRPV1 (1:100, Abcam, Cambridge, UK, ab3487), and rabbit anti-H1R (Histamine receptor 1, 1:100, Abcam, Cambridge, UK, ab 75236). Secondary antibodies were used as follows: goat anti-mouse (FITC, 1:300, Abcam, Cambridge, UK, ab97229), pig anti-rabbit (TRITC, 1:300, Dako, Agilent, Santa Clara, CA, USA R0156), goat anti-mouse (TRITC, 1:300, Abcam, Cambridge, UK, ab5928), goat anti-rabbit (Chromeo488, 1:300, Abcam, Cambridge, UK, ab60314), and goat anti-mouse (TRITC, 1:300, Sigma-Aldrich, Saint-Louis, MO, USA, T7782). Immunostaining without the addition of a primary antibody was performed as a control.

### 2.4. Reverse Transcription—Quantitative Polymerase Chain Reaction (RT-qPCR)

RNAs was extracted from SKPs at each end of the differentiation step using Tri-reagent (Sigma-Aldrich, Saint-Louis, MO, USA, T9424). Quantification and quality control of total RNAs were performed by measuring optical densities at 260 and 280 nm. Equivalent amounts of total RNA were reverse transcribed using a High Capacity cDNA Reverse Transcriptase kit (Applied Biosystems, Thermo Fisher Scientific, Waltham, MA, USA). The expression of genes involved at each step of sensory neuronal differentiation was quantified on an Applied Biosystems Real-Time PCR system with SYBR green PCR master mix (Applied Biosystems) and 25 ng of cDNA per 20 µL PCR. Hybridization and elongation were realized at 60 °C. A list of probes is indicated in Table 1. The 2^−ΔΔCt^ method was used with β-ACTIN as an internal control to relatively quantify the detected transcripts. 

### 2.5. Intracellular Ca^2+^ Measurement

The differentiated SKPs (up to > 15 days with maturation medium) were seeded on X-well (Sarstedt) loaded with 4 µM Fura 2-AM (Molecular Devices, Sunnyvale, CA, USA) and 2 µM pluronic acid (Thermo Scientific, Waltham, MA, USA) in the dark for 45 min at 37 °C and 5% CO_2_. The cells were washed to remove the excess dye, and intracellular Ca^2+^ measurements were performed according to the protocol described by Sakka et al. [28]. For the data analysis, the F340/F380 ratio of emitted fluorescence signals intensity recorded at 340 and 380 nM excitation wavelengths was calculated for each measurement time point. The amplitude of the elicited Ca^2+^ responses was measured by calculating the difference between the basal and maximal F340/F380 ratio values. Cells were stimulated with capsaicin at 10 µM (Sigma, M2028), SLIGKV at 50 µM (PAR2-AP, Sigma, S9192), polygodial at 3 µM (Santacruz, sc-201489), and histamine at 1 mM (Sigma, H7125) to evaluate the functionality of the receptor and the capacity of differentiated cells to induced a Ca^2+^ signal following stimulation. For each agonist, a random field was recorded. Each activation by an agonist was recorded in a compartment that was distinct from other activations.

### 2.6. Patch-Clamp

Ionic currents were recorded in a cell-attached configuration of the differentiated SKPs (up to > 15 days with maturation medium) using a macropatch clamp technique at RT (22 ± 2 °C). The pipette (Clark electromedical glass, Holliston, MA, USA) 1.5 mm in diameter, were pulled to obtain 3 µm at the tip, then filled with 22 µm filtered culture medium. Positioning of the pipette was ensured by a micromanipulator (Narishige) and was monitored with an inverted microscope (Olympus IX 70, Olympus, Tokyo, Japan) that was equipped with Hoffman contrast and a progressive-scan digital camera (XC8500CE, Sony, Tokyo, Japan). Patch-clamp experiments were performed in culture medium. The transient currents (fast inactivating potassium or sodium currents) were visualized using a protocol in which 20-ms pulses were used, ranging from −40 to + 100 mV from a hyperpolarized holding potential of −100 mV. It should be noted that a fast sodium current was not observed if the membrane was not hyperpolarized to −100mV before applying test pulses. A 3-s interval between each test pulse was observed to ensure the complete recovery of the sodium channel from inactivation. The current-voltage relationship corresponding to the long-lasting currents (potassium or calcium) was measured according to another protocol with no preliminary hyperpolarization and corresponding to a cycle of 100-ms test pulses imposing a membrane potential ranging from −100 to +100 mV in 10-mV increments. Cesium chloride was added to suppress the potassium current and reveal the calcium current. All stimulation protocols were repeated in triplicate for each patch to ensure current stability. All patches with unreliable current amplitudes were discarded. 

### 2.7. Statistical Analysis

The statistical significance in mean values among multiple sample groups was analyzed with the Kruskal–Wallis test. The Mann–Whitney test was used to examine the statistical significance between the two sample groups. The significance level was defined as *p* < 0.05, and significance tests were conducted using Prism6 (GraphPad Software, Inc., San Diego, CA, USA).

## 3. Results

### 3.1. Characterization of Human SKPs

**Figure 2:**
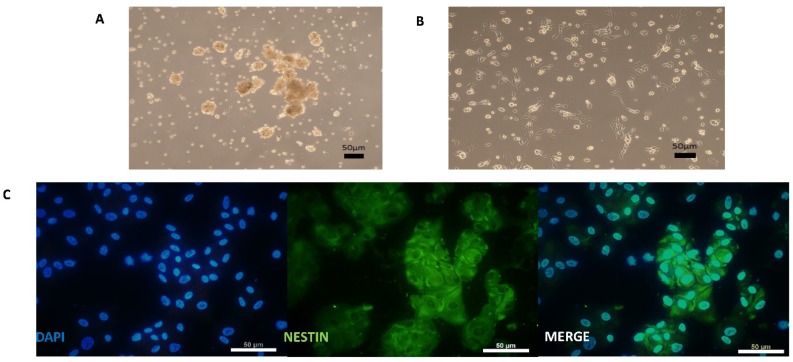
Cultured human SKPs under maintenance medium with uncoated support. **A**—Picture of SKPs in neurospheres before adherence. **B**—Picture of SKPs after adherence. **C**—Immunocytochemistry analysis of SKPs with NESTIN (Protein of neural progenitor markers). Scale bars = 50 µm.

SKPs grown in suspension as neurospheres (Figure 2A) and maintained in a proliferative state needed to be dissociated until spontaneous adhesion occurred. Then, they proliferated as adherent cells on an uncoated plate for a few days (Figure 2B).

**Figure 3:**
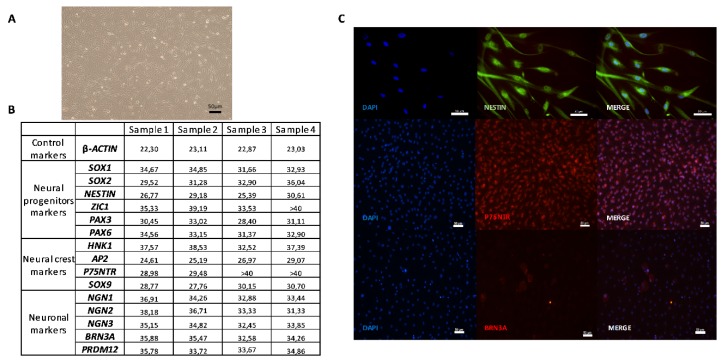
Characterization of human SKPs on coating Poly-l-ornithine/Laminin/Fibronectin (Day 0). **A**—Picture of proliferating SKPs with maintenance medium. **B**—Table of mean cycle thresholds (Ct) for each sample during the undifferentiated SKPs phase (in the maintenance medium); of a non-exhaustive list of genes present at different stages of differentiation of neural crest stem cells (NCSCs) into sensory neurons (SNs). The results were obtained in RT-qPCR. **C**—Immunocytochemistry analysis of undifferentiated SKPs (in the maintenance medium and coated plate) with one protein for each step of differentiation of NCSCs into SNs: NESTIN (Protein of neural progenitor markers); P75NTR (Protein of neural crest markers); BRN3A (Protein of Neuronal markers). Scale bars = 50 µm.

These cells expressed NESTIN (Figure 2C). Before differentiation induction on a coated plate, the SKP cells were examined for the expression of markers of neural progenitor cells, neural crest cells, and SNs to establish a baseline characterization. As indicated in Figure 2C and Figure 3C, the cells were positive for the neural progenitor markers NESTIN and P75NTR but not BRN3A, which is a sensory neuronal marker. Different genes specifically expressed only in a stem cell state and not in differentiated cells were screened to characterize the undifferentiated SKPs isolated from the different patients (Figure 3B). Their phenotype was determined by examining genes corresponding to different stages in the development of NCSCs. In this study, we considered that cycle thresholds (Ct) greater than 36 showed an insufficient amount or absence of the gene of interest. For example, the expression of neuronal markers was very weak or not detectable, with Ct values greater than 36, in the patient SKP cultures prior to the initiation of the differentiation protocol. All studied samples globally expressed *SOX9* in premigratory or migratory neural crest-derived cells. These cells also expressed *AP2*, another neural crest progenitor marker. *P75NTR* was expressed in two samples and not expressed in two other samples (Figure 3B,C). The expression of *HNK1*, another marker of NCSCs, was low or absent in undifferentiated SKP populations. In summary, (Figure 3B), SKP populations from different patients displayed variability in gene expression. The results from immunostaining and RT-qPCR confirmed that these cells contained a mixed population of neural progenitors and neural crest (Figure 3B,C).

### 3.2. Induction of Neurogenesis

**Figure 4:**
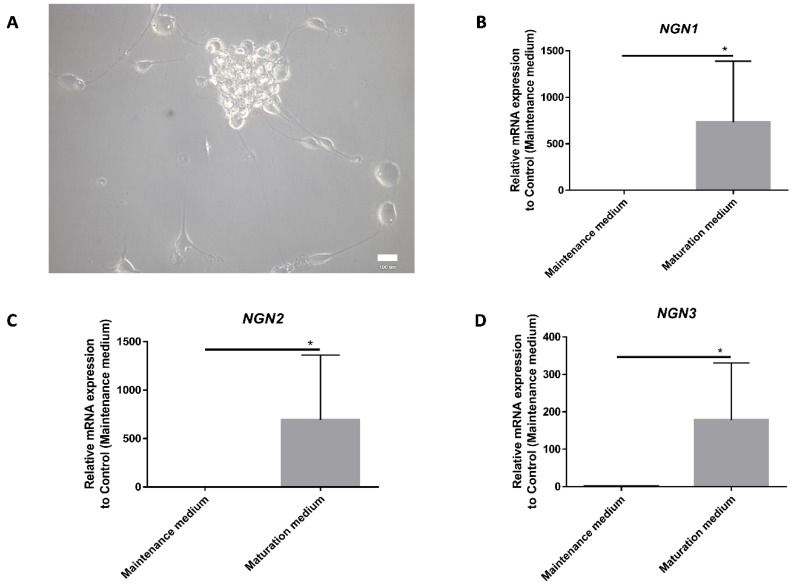
Induction of differentiation into neurons regulated by Neurogenins. **A**—Picture of differentiated SKPs into neuron-like cells after the maturation step using phase-contrast microscopy. **B**, **C,** and **D**—Results obtained with RT-qPCR and normalized by the control (Maintenance medium). The differential of Neurogenins (*NGN*) 1, 2, and 3 are analyzed between SKPs in maintenance medium and differentiated SKPs in maturation medium. Graphic represent the mean +/− SEM, * *p* < 0,05. Scale bar = 100 µm, *n* = 4.

SKP cells were induced to generate SNs-like by treatment with trophic/growth factors (GDNF, BDNF, BMP4, and NGF), chemical factors, and small molecules (purmorphamine, CHIR99021). At the end of the differentiation process, the cells acquired a neuronal-type morphology with cell bodies and neurites (Figure 4A), and they tended to form clusters with their cell bodies, while their axons were engaged with others. We confirmed the potential of the differentiation protocol to induce neurogenesis of neural crest progenitors (SKPs) by measuring the expression of the *NGN*s. The amount of *NGN1*, *2*, and *3* mRNA increased significantly from undifferentiated SKPs in the maintenance medium to SKPs in the maturation medium (Figure 4B,D). The increase in the expression of *NGN1* and *2* tended to be greater than the increase in *NGN3*; they could be more highly expressed by differentiated SKPs in the maturation medium than by the undifferentiated SKPs in the maintenance medium.

### 3.3. Analysis of the SKPs During Differentiation

**Figure 5:**
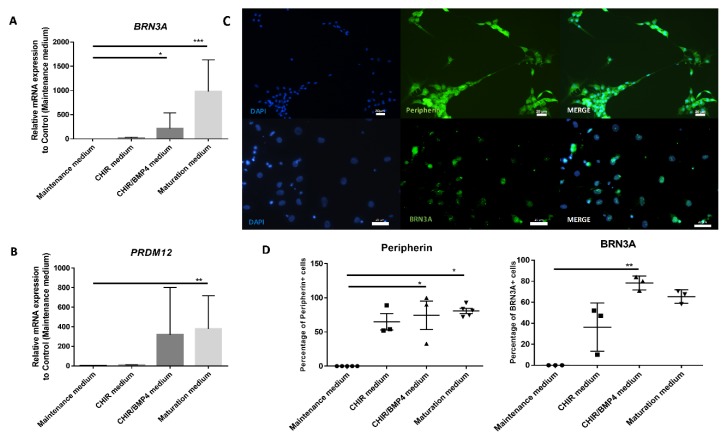
Induction of the expression of SN markers. Temporal gene expression analysis comparing each step of the differentiation process: (**A**) *BRN3A* and (**B**) *PRDM12*. **C** and **D**: Picture of immunocytochemistry (**C**) and analysis comparing the percentage of PERIPHERIN- and BRN3A-positive cells between SKPs in maintenance medium and each step of differentiation (**D**). Graphic represents the mean +/− SEM. * *p* < 0.05; ** *p* < 0.005; *** *p* < 0.0005, *n* = 6 for RT-qPCR and *n* = 3 for immunocytochemistry (*n* = 5 for control and maturation medium of peripherin). Scale bar = 20 µm for PERIPHERIN and 50 µm for BRN3A.

To confirm that SKPs differentiated into SNs-like, cultures were either fixed and immunostained for a SNs marker (BRN3A) or a marker for peripheral neurons (PERIPHERIN), or they were lysed to quantify the amount of *BRN3A* mRNA or a marker of pain-sensing nerve cells (*PRDM12*). The analysis of the mRNA expression of *BRN3A* and *PRDM12* genes revealed a progressive increase during the differentiation protocol and showed a significant difference from the undifferentiated cells at the end of the process (Figure 5A,B). The incubation of cells in the maturation medium resulted in increased expression levels of the *BRN3A* and *PRDM12* genes, reaching increases of 1000 times for *PRDM12* and 2500 times for *BRN3A* compared to the expression by cells grown as a control (maintenance medium).

The same kinetics were analyzed by immunocytochemistry, and a concomitant, gradual increase in BRN3A and PERIPHERIN protein expression was also observed during differentiation (Figure 5C,D). No BRN3A or PERIPHERIN was found in cells grown in the maintenance medium. This upward trend in expression began two days after the application of CHIR99021 alone (37% and 65% increase for BRN3A and PERIPHERIN, respectively) and then increased further with the addition of BMP4 (78% and 75%, respectively) to reach a plateau with the maturation medium (65% and 83%, respectively). The number of PERIPHERIN-expressing cells (approximately 75%–80%) and BRN3A-expressing cells (between 65% and 75%) did not significantly increase with the addition of maturation medium (Figure 5D).

### 3.4. Functional Analysis of SNs-Like Issued From SKPs

**Figure 6:**
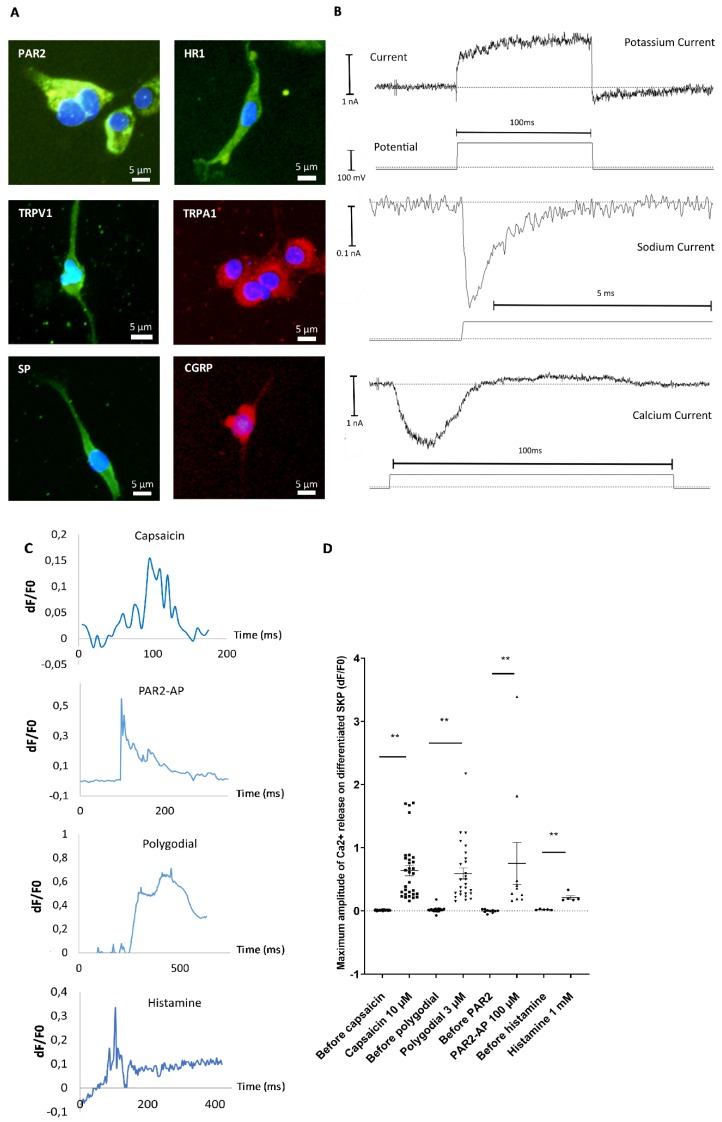
Descriptive and functional analysis of SKPs differentiated into SNs after 15 days of maturation. **A**—Immunocytochemistry analysis of substance P (SP), calcitonin gene-related peptide (CGRP), transient receptor potential vanilloid (TRPV1), transient receptor potential ankyrin (TRPA1), histamine receptor 1 (HR1), and protease-activated receptor 2 (PAR2) (*n* = 6). **B**—Patch-clamp analysis of the presence of sodic, potassic, and calcium channels on random cells. Graphs are representative of responding cells. **C**—Analysis by calcium imaging of the response of SNs to capsaicin, polygodial, PAR2-AP, or histamine. Results are expressed in delta F/F0 in the function of time expressed in milliseconds. Graphs are representative of responding cells in a manipulation of three independent experimentation of differentiation. **D**—Dot plots represent maximum amplitude of all responding cells after agonist application of three independent experiments (two for histamine) of differentiation, one dot corresponding to one cell. ** *p* < 0.01. The Bars represent the mean +/− SEM.

To investigate the functionality of cells obtained after differentiation, we analyzed some major receptors and neuropeptides implicated in inflammation, pruritus, or pain. Immunocytofluorescence confirmed the expression of substance P (SP), calcitonin gene-related peptide (CGRP), histamine receptor 1 (HR1), transient receptor potential ankyrin (TRPA1), transient receptor potential vanilloid (TRPV1) and protease-activated receptor 2 (PAR2) (Figure 6A). 

To validate the functionality of ion channels at the cell surface, patch clamp experiments were conducted. The data indicated the presence of different types of currents corresponding to long-lasting and transient currents. Evidence of transient calcium and sodium currents was observed, as well as long-lasting potassium currents, indicating functional ionic exchange and excitability of the differentiated cells (Figure 6B). To more precisely analyze functionality, calcium signals induced by different agonists were evaluated using a calcium imaging approach. At the end of the maturation process, cells were incubated with a specific agonist of each of the following receptors of interest; the response was analyzed using calcium imaging: capsaicin for TRPV1, polygodial for TRPA1, PAR2-AP (agonist peptide) for PAR2, and histamine for the HR1. The responsiveness of differentiated cells for calcium signal induction was evaluated for each experimental condition. Cells that were positive for calcium movement were recorded (e.g., Figure 6A). A total of 52% of the differentiated cells responded to capsaicin, 81% to polygodial, 17% to PAR2-AP, and 33% to histamine. The Ca^2+^ signals amplitude in differentiated cells were always largely increased compared to without agonist (mean amplitude Ca^2+^ signals: 0.64 for capsaicin, 0.59 for polygodial, 0.75 for PAR2-AP, and 0.21 for histamine stimulations versus 0.01, 0.015, 0.0, and 0.02 for the respective controls—Figure 6D). These data indicate that in our protocol, SKPs had acquired some key functionality of SNs-like.

## 4. Discussion

Working with human stem cells differentiated into a cellular type of interest, such as SNs, is a valuable method for in vitro studies on human biology that offers an alternative to cells isolated from animals and to cell lines. In the literature, several studies have shown the differentiation of pluripotent stem cells (hESCs and hiPSCs) into SNs without functionality demonstration [2,4,5,29,30]. However, differentiation from SKP multipotent stem cells with NCSC characteristics has never been reported. In this study, we have shown that the differentiation of SKPs into SNs is feasible. For this process, we used and adapted the protocol elaborated by Reinhardt’s team in 2013 [5]. Interestingly, because SKPs are more engaged in the specification process than pluripotent stem cells, the number of steps for differentiation into SNs was reduced. In our study, we provided many arguments showing the differentiation of SKPs into neuronal cells, such as the expression of canonical peripheral markers, such as BRN3A, PERIPHERIN, and PRDM12, in SNs derived from SKPs.

SKPs are a population of stem cells that include various stem cell subpopulations, in particular, NCSCs [16,31,32]. The characterization of this cell population at the undifferentiated step was mainly performed by RT-qPCR. A large number of genes were screened, each representing a step in the differentiation process of these precursors (Figure 3B). Proportions of markers of NCSCs are variable. This finding could reflect either a natural variability among donors, or it could be linked to the process of cell selection. RT-qPCR showed that undifferentiated SKPs expressed the same transcription factor genes present in a diverse, in vivo population of NCSCs. As expected, the undifferentiated SKPs expressed NESTIN, a neural precursor marker, and its expression was preserved during expansion with FGF and EGF [12,13,33]. All the genes studied were differentially expressed according to the donors, with inter- and intra-population variability among the different samples [14]. This observation may be explained by the heterogeneity of the cells within the same sample, which suggests that cells could be taken at different stages of commitment.

During SKP differentiation, morphological changes were observed. Cells acquired a neuron-like morphology, with a clear segmentation of the cell body and neurites (Figure 4A). They formed clusters similar to those observed in rat embryonic SN cell cultures, in which they formed groups whose axons developed a network [2]. The network of differentiated SKPs was not as developed as the one formed by the embryonic SN cell rats, but they resembled one another. Neuronal phenotype observation has been confirmed by the verification of PERIPHERIN expression induction. 

This process of neurogenesis leads to the formation of the sensory nervous system, which is composed of a variety of types of SNs enabling the sensation of touch (low-threshold mechanoreceptor neurons), temperature (thermal SNs), pain (nociceptive neurons), movement, and spatial position (proprioceptive neurons). Each function is characterized by its own unique set of receptors and ion channels, and their differentiation depends on a unique set of transcription factors. NGN1 and 2 control sensory neurogenesis, while NGN3 is involved in the development of the central nervous system [23,34]. Our results show a greater increase in NGN1 and 2 in NCSC differentiation, which suggests the possibility of SKP to express NGN after neuronal induction. Furthermore, the pathway of differentiation for these cells is in favor of a sensory specification (Figure 4B,D). Some other markers of SNs were induced during differentiation, such as BRN3A, an SN marker, PERIPHERIN, a peripheral neuron marker, and PRDM12, a pain-sensing nerve cell marker. Furthermore, neuropeptides (SP and CGRP) expressed by SNs were equally detected. The upregulation of the neuron-specific transcriptional regulators *BRN3A* and *PRDM12* is involved in the transition from neurogenic progenitors into SNs. However, the induction of the BRN3A marker did not correspond to 100% of the differentiated cells, being present in approximately 70% of them; these results suggest that not all cells were specified to begin sensory differentiation, but some potentially entered into the central pathway. This result could be traced back to the role of the transcription factor NGN3. These data do not exclude the presence of a small proportion of nonneuronal cells.

Upon differentiation, from the application of CHIR99021, a GSK3β inhibitor, markers of SNs such as BRN3A, PERIPHERIN, or PRDM12 began to appear, and, with the addition of BMP-4, there was significant induction for two of them: BRN3A and PERIPHERIN (Figure 5). The GSK3β inhibitor activates the Wnt/B-catenin pathway and allows the acquisition of the SN profile that is mediated by NGN1 and 2 [23,24]. The addition of BMP-4 acts as a regulator of sensory neuronal differentiation during the maturation of SNs [25]. When NGF, GDNF, and BDNF were added to the maturation medium, there was increased *BRN3A* and *PRDM12* transcriptional activity. Indeed, the upstream use of BMP-4 enabled the acquisition of neuronal dependence on NGF and BDNF for their survival [26].

The functionality of SNs from SKPs was evaluated after the end of the maturation step. The cells possessed a panel of sodium, calcium, and potassium channels, the activities of which were demonstrated by patch-clamp analysis. Cells express receptors present in peripheral SNs, such as TRPV1, TRPA1, PAR2, and H1R. These receptors were functional in differentiated cells, as demonstrated by the Ca^2+^ signals recorded following stimulation with their specific agonist. TRPV1 is widely expressed in skin SNs and has a prominent role in pain sensation and inflammation, mainly via several locally released neuropeptides. TRPA1 is also expressed in SNs and is implicated in pain and inflammation as well. H1R is the main receptor for pruritic sensation of the histamine-dependent pruritic pathway. PAR2 is known to be implicated in neurogenic inflammation and pruritus. Taken together, these data show that we obtained functional SNs from the peripheral nervous system. These neurons would be very interesting to apply to in vitro studies on pain, pruritus, neurogenic inflammation, and others.

## 5. Conclusions

From SKPs, we obtained differentiated cells that were induced to differentiate by activation of specific pathways, which we characterized here. These cells expressed different markers of SNs (including transcription factors, cytoskeleton members, channel receptors, and neuropeptides), and further studies showed that they could respond to specific agonists or antagonists and electrical activation. This work demonstrated the feasibility of obtaining functional SNs from a source other than iPSCs or hESCs, which is an important step that avoids ethical considerations or the use of genetically modified organisms. Furthermore, the process to obtain SNs is shorter, as SKPs are already NCSC cells. In conclusion, this work demonstrates that routine in vitro production of functional SNs-like directly from in vivo adult precursor cells is possible and that the cells obtained with our protocol could be a good model for pharmacological studies in the pain, pruritus or inflammation domains.

## Figures and Tables

**Table 1:** List of primers used in the study.

Gene	Forward 5′->3′	Reverse 5′->3′
HNK-1	GCT GAC GAC GAC AAC ACC TA	CGG TGT ACC AGC CAA CAA C
p75NTR	GTC CCC CGC AGA GCC GTT GAG AAG	TGA ACC ACA CGC CCC CAC CAG AG
NESTIN	CTC CAG AAA CTC AAG CAC C	TGA TTC CTG ATT CTC CTC TTC C
BRN3A	CGT ACC ACA CGA TGA ACA GC	AGG AGA TGT GGT CCA GCA GA
Pax6	AGT GAA TCA GCT CGG TGG TGT CTT	TGC AGA ATT CGG GAA ATG TCG CAC
Pax3	TAC CAG CCC ACG TCT ATT CCA CAA	TTT GGT GTA CAG TGC TCG GAG GAA
Sox1	GGC TTT TGT ACA GAC GTT CCC	AAC CCA AGT CTG GTG TCA GC
Sox9	ACG GCT CCA GCA AGA ACA AG	TTG TGC AGA TGC GGG TAC TG
Zic1	AAA CTG GTT AAC CAA ATC CGC	CTC AAA CTC GCA CTT GAA GG
Sox2	GCA CAT GAA CGG CTG GAG CAA CG	TGC TGC GAG TAG GAC ATG CTG TAG G
AP2	TCT TGT CAC TTG CTC ATT GGG	GTT ACC CTG CTC ACA TCA CTA G
Ngn1	CAA-CCG-CAT-GCA-CAA-CTT-GA	GCG-TCT-CGA-TTT-TGG-TGA-GC
Ngn2	TGG-GTC-TGG-TAC-ACG-ATT-GC	GTC-TTC-TTG-ATG-CGC-TGC-AC
Ngn3	CAA-ACA-CCA-CAG-GAG-TCT-ATC-C	GGT-CTG-GGA-TCC-TTG-ATT-CTT-C
PRDM12	CAG-GTT-CTG-CTC-CTG-TTC-GT-3’	TGT-GGG-AGG-TGT-TCA-ATG-AGG
β-actin	GAG ACC TTC AAC ACC CCA GC	ATG TCA CGC ACG ATT TCC CT
